# Attitudinal changes of undergraduate students learning online interprofessional education for patient safety: Comparative evaluation of an online program using the DID method

**DOI:** 10.3205/zma001696

**Published:** 2024-09-16

**Authors:** Shinjiro Nozaki, Takatoshi Makino, Bumsuk Lee, Hiroki Matsui, Ena Sato, Hiromitsu Shinozaki, Hideomi Watanabe

**Affiliations:** 1 Gunma University, Graduate School of Health Sciences, Gunma, Japan; 2 Gunma University, WHO Collaborating Centre for Research and Training on Interprofessional Education, Gunma, Japan; 3 Takasaki University of Health and Welfare, Gunma, Japan

**Keywords:** health-care professions, interprofessional education, patient safety, difference-in-differences analysis, online program

## Abstract

**Objective:**

Interprofessional education (IPE) can cultivate competencies in multidisciplinary collaboration for patient safety, and both face-to-face and online IPE programs have recently been introduced. This study aimed to elucidate the effects of the online IPE program on undergraduate students after the coronavirus disease 2019 pandemic.

**Methods:**

The difference-in-differences method was used to assess undergraduate students in the Schools of Medicine and Health Sciences and in the Faculty of Pharmacy at Takasaki University of Health and Welfare who participated in IPE programs at Gunma University (face-to-face IPE was implemented in 2019 and online IPE in 2020). We distributed a questionnaire that included modified versions of the Attitudes Toward Health Care Teams Scale (ATHCTS) and the Teamwork Attitudes Questionnaire (T-TAQ) to evaluate attitudes toward health-care teams and collaboration for patient safety, respectively, and then compared the differences.

**Results:**

The mean score on the “team efficiency” subscale of the ATHCTS in the online IPE program was significantly lower than that in the face-to-face IPE program. Scores on the T-TAQ in two categories, “mutual support” and “communication”, were significantly higher in the online IPE program, which suggests that it may have a similar effect on students learning collaborative practice for patient safety. However, due to technological difficulties, the online IPE program reduced the educational effects for “team efficiency”. The improvements in “mutual support” and “communication” seen in the online IPE program, suggest its necessity for collaborative practice for patient safety.

**Conclusion:**

These findings suggest that an online IPE program for patient safety may provide better education effects as a whole, but efforts are needed to minimize the associated technological difficulties.

## Zusammenfassung

**Ziel:**

Interprofessionelle Ausbildung (IPE) kann Kompetenzen in der multidisziplinären Zusammenarbeit für die Patientensicherheit aufbauen, und in jüngster Zeit wurden sowohl Präsenz- als auch Online-IPE-Programme eingeführt. Das Ziel dieser Studie war es, die Auswirkungen des Online-IPE-Programms auf Studenten nach der Coronavirus-Pandemie 2019 zu untersuchen.

**Methoden:**

Der Differenz-von-Differenzen-Ansatz wurde zur Beurteilung von Studenten der Medizin- und Gesundheitswissenschaften sowie der Pharmazie-Fakultät an der Takasaki Universität für Gesundheit und Soziales angewendet, die an IPE-Programmen der Universität Gunma teilgenommen haben (Präsenz-IPE wurde 2019 und Online-IPE 2020 implementiert). Wir verteilten einen Fragebogen, der modifizierte Versionen der Attitudes Towards Health Care Teams Scale (ATHCTS) und des Teamwork Attitudes Questionnaire (T-TAQ) enthielt. Die Ergebnisse der Studie sollten dazu genutzt werden, die Einstellungen gegenüber Gesundheitspflegeteams und die Zusammenarbeit für die Patientensicherheit zu bewerten und dann die Unterschiede zu vergleichen.

**Ergebnisse:**

Der Mittelwert der Subskala „Teameffizienz“ des ATHCTS war im Online-IPE-Programm signifikant niedriger als im Präsenz-IPE-Programm. Die Ergebnisse des T-TAQ in zwei Kategorien, „gegenseitige Unterstützung“ und „Kommunikation“, waren im Online-IPE-Programm signifikant höher. Dies deutet darauf hin, dass es einen ähnlichen Effekt auf die Studierenden haben könnte, die kollaborative Praxis für die Patientensicherheit lernen. Aufgrund technologischer Schwierigkeiten verringerte das Online-IPE-Programm jedoch die pädagogischen Auswirkungen auf die „Teameffizienz“. Die Verbesserungen in „gegenseitiger Unterstützung“ und „Kommunikation“ im Online-IPE-Programm deuten auf die Notwendigkeit einer kollaborativen Praxis für die Patientensicherheit hin.

**Fazit:**

Diese Ergebnisse deuten darauf hin, dass ein Online-IPE-Programm für Patientensicherheit insgesamt bessere Ausbildungseffekte erzielen kann, aber es müssen Anstrengungen unternommen werden, um die damit verbundenen technischen Schwierigkeiten zu minimieren.

## 1. Introduction

Useful communication that underpins well-functioning teamwork plays an important role in safe health care, including patient safety [1]. Vincent described organizations with a positive safety culture as being “characterized by communications founded on mutual trust, by shared perceptions of the importance of safety, and by confidence in the efficacy of preventative measures” [2]. To train for interprofessional collaboration during university, interprofessional education (IPE) is needed for all health-care professions, and such an approach is provided for in the drafts of new medical licensing regulations [3]. The culture of patient safety of a given group or institution is shaped by the individual attitudes of its healthcare workers. The attitude toward collaborative practice must be fostered through an educational approach, namely, IPE programs, which have “a significant capacity to cultivate collaborative practice competencies to collaborate for patient safety” [4]. Not only prevention, but also active constructive error management, contributes to patient safety [5].

The World Health Organization declared the coronavirus disease 2019 (COVID-19) outbreak a pandemic on March 11, 2020 [6]. On April 11, 2020, the Prime Minister of Japan declared a nationwide state of emergency on the basis of the Act on Special Measures for Pandemic Influenza and New Infectious Diseases Preparedness and Response [7]. Lectures were rapidly developed to be delivered online through Zoom, because technologically advanced approaches are known to be able to increase engagement among medical students [8]. A growing number of colleges and universities are transitioning from traditional face-to-face to online teaching methods or a hybrid of both [9]. However, students sometimes find it more difficult to concentrate and participate during online lessons such as those conducted through Zoom [10], [11].

Meta-analyses on studies of the effects of e-learning have found that online education can improve various professional competencies, including attitudes, knowledge, skills, and behaviors, and have reported that online learning “can be as effective as physical attendance in a traditional classroom” [12], [13]. Consequently, virtual or distance learning has become one of the most important types of educational modalities, and information technology has created many opportunities for education [14]. Before the COVID-19 pandemic, the Internet was an essential and useful tool for distance learning, but played only a supplementary role in traditional and conventional classes [14]. Some studies have reported finding no differences in performance by modality (e.g., online vs. face-to-face courses) [15], [16]. In addition, no differences in academic outcomes were reported between face-to-face and online learning [17], [18]. At the present stage of the pandemic, the roles of face-to-face and online learning may be comparable, but the means by which the outcomes for learners are attained require distinct forms of expertise [19]. Although many studies evaluated the effects of IPE between online and face-to-face learning before the pandemic [20], [21], [22], [23], few have evaluated the effects of IPE in fostering a collaborative culture for patient safety comparing between the periods before and after the start of the pandemic.

The difference-in-differences (DID) method has a long history in disciplines outside of epidemiology [24] and can be applied to “any model where outcomes are observed in a minimum of two groups at different time points, assuming confounders are time invariant” [25], [26]. The DID method yields impartial effect estimates if the trend over time would have been the same between the intervention and comparison groups [27]. Given this background, with the aim of developing a curriculum that can foster a collaborative culture for patient safety, the present study examined the effects of an IPE program for undergraduate students before and after the start of the COVID-19 pandemic using the DID method.

## 2. Methods

### 2.1. Study design

The DID method was used to evaluate students who participated in an IPE program at Gunma University that implemented face-to-face IPE in 2019 and online IPE in 2020.

### 2.2. Study population

The Gunma University Faculty of Medicine consists of the School of Medicine (GUSM, enrollment: 120 students) and Gunma University School of Health Sciences (GUSHS, enrollment: 160 students), including the departments of Nursing (NS, 80 students), Laboratory Sciences (LS, 40 students), Physical Therapy (PT, 20 students), and Occupational Therapy (OT, 20 students) [4], while Takasaki University of Health and Welfare the Faculty of Pharmacy (TUHWFP, enrollment: 90 students) consists of the department of Pharmacy. Gunma University has a credit transfer system for students at TUHWFP. The current IPE program at Gunma University has implemented mandatory subjects for third-year students in GUSHS and elective subjects for fourth-year students in GUSM and fifth-year students in TUHWFP. In total, 22 (14 in 2019 and 8 in 2020) of 240 students in GUSM, 315 (162 in 2019 and 153 in 2020) of 315 students in GUSHS and 30 (20 in 2019 and 10 in 2020) of 180 students in TUHWFP took this training-style program. We distributed the questionnaire for all registered students. The present study was performed during the 2019 and 2020 academic years.

### 2.3. Study setting

#### 2.3.1. Interprofessional education program at Gunma University

The program was administered to students in the 2019 academic year as described previously [28], and in the 2020 academic year as an online–hybrid process. The points of difference between academic years 2019 and 2020 were as follows:

1. In 2019, face-to-face instruction was provided to brief students on the program and introduce the faculty, whereas in 2020, instruction was provided via Zoom [https://zoom.us/ja/signin#/login]. All handouts were hand-distributed by the faculty in 2019, and online through the Moodle open-source learning management system [https://mdl.media.gunma-u.ac.jp/GU/index.php] in 2020.
2. A sense of unity was promoted in each group through a sports game to acquire preferred training facilities for the face-to-face instruction in 2019, whereas the program committee randomly selected the training facility in 2020.
3. Group-work session prior to clinical training to prepare the training agenda and plan clinical training were conducted face-to-face in 2019, whereas such sessions were conducted through Zoom and Google Docs in 2020.
4. Face-to-face clinical training was conducted at each training facility in 2019, whereas students could not obtain clinical training at any training facility because of the COVID-19 pandemic in 2020. Instead, in 2020, students learned interprofessional collaboration at training facilities through formal letter communications.
5. In 2019, group-work sessions were conducted face-to-face after clinical training to prepare a report on each group’s accomplishments and lessons learned, whereas in 2020, such sessions were conducted via Zoom, Google Docs, and Google Slides.
6. In 2019, clinical practice leaders in the training facilities met face-to-face to give a presentation and discuss teamwork at a debriefing meeting, whereas in 2020, the meetings were held via Zoom.
7. A wrap-up session and a general meeting to prepare the final report were conducted face-to-face in 2019, whereas they were conducted via Zoom and Google Docs in 2020.
8. Evaluation surveys before and after the training were conducted on paper in 2019, whereas they were conducted via Google Forms in 2020.
9. The students encountered three main challenges in 2020 during the transition from traditional to online classes. First, they experienced technical issues when attempting to access Moodle, Google, and Zoom. Consequently, they were given a technical briefing on the online system at the beginning of the semester face-to-face and via Zoom. Second, the home Internet environment was an issue for some students. In response, the university began providing free Internet access via rented pocket Wi-Fi devices. Finally, the need for a PC to access the Internet and print handouts independently was an economic issue for some students. To address this issue, parental support groups from GUSHS subsidized a portion of the cost.

### 2.4. Survey instruments

The 21-item Attitudes Toward Health Care Teams Scale (ATHCTS) has been reported to be able to evaluate clinically-based team training programs and to be used as both a pre- and post-test tool for educational interventions with teams [29]. In the present study, we used a modified 14-item version of the ATHCTS [30] that removed items containing the word “physician” to measure attitudes toward healthcare teams. The present study contain medical students as well as health professional. The previous study reported it might be not feasible to use items with labels targeting only physician either [31]. It must be noted that three items (Nos. 2, 6, and 9) were inverted in the analysis. Responses were provided on a five-point Likert scale, from 1 (strongly disagree) to 5 (strongly agree), with a higher score indicating more positive attitudes toward healthcare teams [31]. This instrument has been shown to have very good validity and reliability [23].

A modified Japanese version of the Teamwork Attitudes Questionnaire (T-TAQ), one of the most frequently used instruments in surveys examining attitudes toward teamwork for patient safety [32], was used to evaluate attitudes toward collaboration for patient safety. This modified T-TAQ included four reverse-coded items (item Nos. 20, 21, and 24 in the mutual support category and No. 30 in the communication category) that were changed to positively worded items with reference to a previous study [33]. In Japan, the concept, methods, and evaluation methods of TeamSTTEPS have been established in Japanese used by the modified T-TAQ [33]. Responses were provided on a five-point Likert scale from 1 (strongly disagree) to 5 (strongly agree), according to the methods of Baker et al. [32]. This instrument has also been shown to have very good validity and reliability [4].

### 2.5. Study procedure

This study was performed in the academic years of 2019 and 2020. During the first term, the professors in charge of each class administered and supervised an attitude survey conducted on the undergraduate students.

### 2.6. Statistical analysis

Data from undergraduate students at Gunma University and Takasaki University of Health and Welfare were analyzed using the Japanese version of IBM SPSS for Windows (version 25.0). This method is convenient for removing all missing data from a dataset.

Exploratory factor analysis was conducted on the scale to examine the underlying constructs of the survey. The suitability of the correlation matrix was determined using the Kaiser-Meyer-Olkin estimate of the sampling adequacy and Bartlett’s Test of Sphericity. Using Kaiser’s criterion, the number of factors retained for the initial solutions and entered into the rotations was determined (eigenvalues >1). The initial factor extractions were performed through principal component analysis. Exploratory factor analysis with varimax rotation was then conducted to define a clearer structure [4], [34], [35]. Next, regression factor scores in the scale were calculated to determine how the resultant factors influenced the difference in student enrollment between 2019 and 2020 at Gunma University and Takasaki University of Health and Welfare [36].

Given that the data were not normally distributed according to the Shapiro-Wilk test, Wilcoxon’s signed rank-sum test was used to analyze independent variables. The level of significance was set at 5% for all tests [23].

The DID method was selected as an approach to allow for comparisons over time between nonrandom populations [37] and for comparing the treatment group before and after the intervention with a control group from a suitably matched comparator control site that did not receive the intervention [27], [38]. As described in greater detail below, applying propensity score methods in the context of DID models is complicated by the fact that there were no longer just two groups (intervention and comparison) [27]; we defined g=1 for online in 2020, g=0 for face-to-face in 2019, t=1 as after the training, and t=0 as before the training. Let Ugt be the mean of an outcome variable in group g at time t. We calculated the difference in the means between post and pre in online in 2020 (B=U11–U10), between post and pre in face-to-face in 2019 (A=U01–U00). We calculated the DID (C=B–A). 

This study was approved by the Gunma University Ethics Review Board for Medical Research Involving Human Subjects (No. HS2016-107). Survey responses were kept confidential, and names and other forms of identifying information were removed for analysis. Written informed consent was obtained from all participants for publication of the results. 

## 3. Results

### 3.1. Demography of responding sample

The survey was completed by 315 of 367 students at Gunma University and Takasaki University of Health and Welfare, for an overall response rate of 85.8%; 172 (87.8%) and 143 (83.6%) students completed the survey in 2019 and 2020, respectively (see table 1 (Tab. 1)).

### 3.2. Attitudinal changes according to the modified ATHCTS

The Kaiser–Meyer–Olkin index was 0.925, indicating sampling adequacy, and the Bartlett Sphericity Chi-Square index was 3633.26 (p<.001), convincingly rejecting the null hypothesis that the correlation matrix was an identity matrix and thus unsuitable for factor analysis. Cronbach’s alpha for 14 items was 0.775, revealing a high rate of internal consistency. The modified ATHCTS questionnaire was categorized into three subscales, “quality of care delivery”, “patient-centered care”, and “team efficiency”, with Cronbach’s alpha measures of 0.878, 0.822, and 0.479, respectively. The factor solutions corresponded well to those reported in a previous study [32].

**Figure 1:**
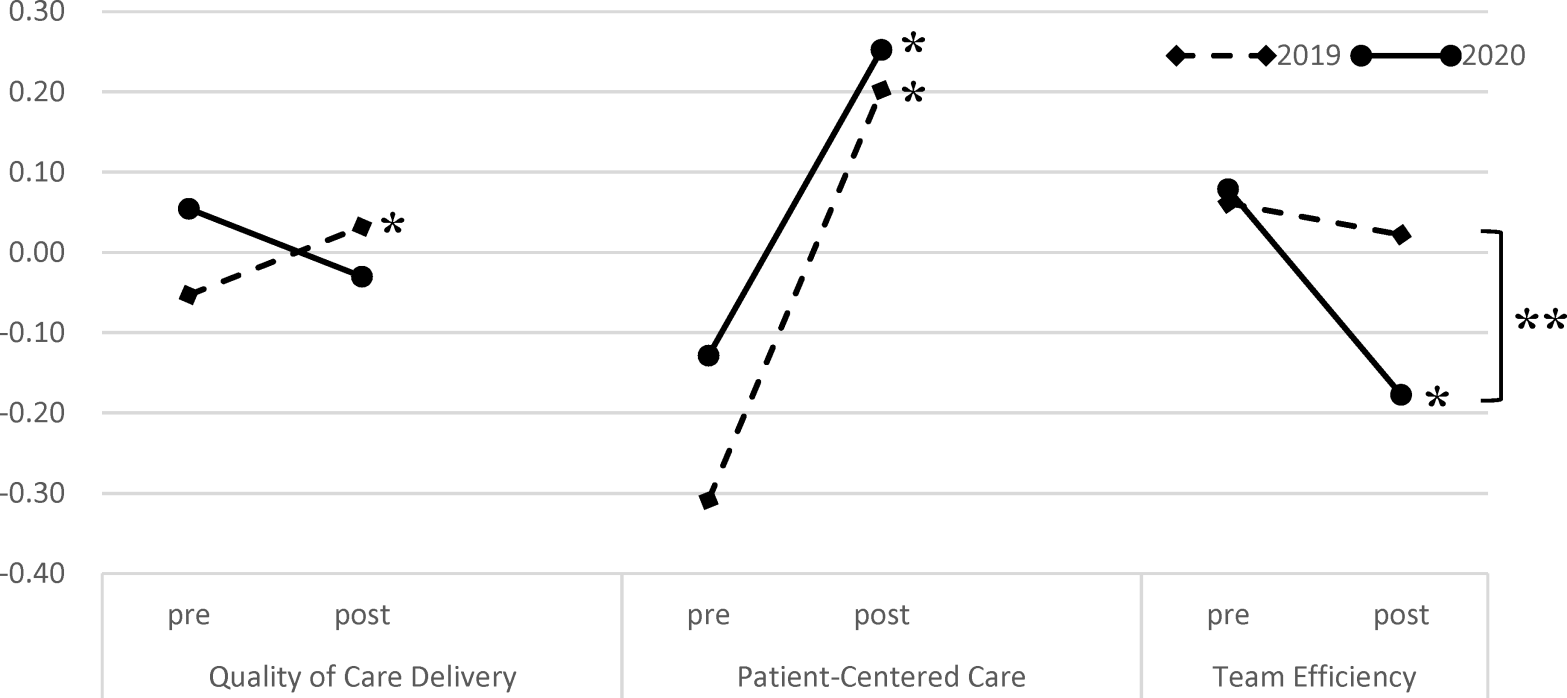
Comparison of regression factor scores of the modified ATHCTS between 2019 and 2020

As shown in figure 1 (Fig. 1), in 2019, the regression factor scores for “quality of care delivery” and “patient-centered care” were significantly increased after training (-0.053±0.750 vs. 0.032±0.965, *Z*=-1.984, *p*=.47 and -0.308±0.798 vs. 0.202±0.879, *Z*=-6.795, *p*<.001; respectively), whereas minimal change was seen for “team efficiency” (0.061±0.697 vs. 0.022±0.941, *Z*=-0.722, ns). Meanwhile, in 2020, the regression factor scores for “patient-centered care” were significantly increased (-0.129±0.722 vs. 0.252±0.854, *Z*=-5.082, *p*<.001), whereas those for “team efficiency” were significantly decreased after training (0.079±0.690 vs. -0.177±0.736, *Z*=-4.053, *p*<.001). In addition, in 2020, the regression factor scores for “team efficiency” were significantly lower than those in 2019 compared with the post-IPE stage.

### 3.3. Attitudinal changes according to the T-TAQ

**Figure 2:**
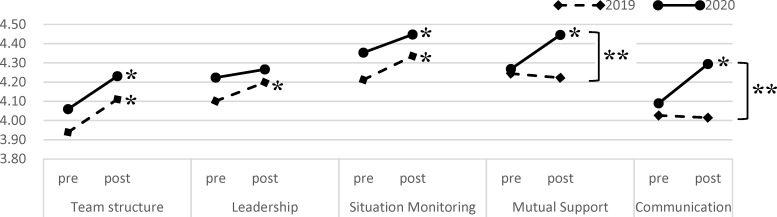
Comparison of mean score of the T-TAQ between 2019 and 2020

As shown in figure 2 (Fig. 2), in 2019, the mean scores for “team structure”, “leadership”, and “situation monitoring” were significantly increased after training (3.939±0.419 vs. 4.110±0.520, *Z*=-5.405, *p*<.001, 4.101±0.501 vs. 4.199±0.553, *Z*=-3.049, *p*=.002, and 4.213±0.447 vs. 4.335±0.547, *Z*=-4.299, *p*<.001; respectively), whereas no significant change was seen in student attitudes in terms of “mutual support” or “communication” (4.245±0.467 vs. 4.223±0.461, *Z*=-0.376, ns, and 4.026±0.512 vs. 4.015±0.535, *Z*=-0.018, ns, respectively). In 2020, the mean scores for “team structure”, “situation monitoring”, “mutual support”, and “communication” were significantly increased (4.059±0.396 vs. 4.231±0.448, *Z*=-4.168, *p*<.001, 4.353±0.420 vs. 4.448±0.442, *Z*=-2.802, *p*=.005, 4.268±0.451 vs. 4.445±0.447, *Z*=-4.658, *p*<.001, and 4.090±0.399 vs. 4.294±0.442, *Z*=-5.446, *p*<.001; respectively), whereas no significant change was seen in student attitudes in terms of “leadership” (4.224±0.427 vs. 4.266±0.423, *Z*=-1.501, ns). In addition, the mean scores for “mutual support” and “communication” at the post-IPE stage were significantly higher in 2020 than in 2019.

### 3.4. Comparative evaluation between the two academic years using the DID method

As shown in table 2 (Tab. 2), the results of the DID analysis indicated that “team efficiency” was associated with significantly lower scores in the online than in the face-to-face IPE program (-0.247; 95% confidence interval [CI], -0.354 to 0.047; *Z*=-2.454, *p*=.014). The DID analysis also revealed no significant differences between face-to-face and online IPE in either “quality of care delivery” or “patient-centered care” (-0.161; 95% CI, -0.412 to 0.106; *Z*=-1.122, ns, and -0.135; 95% CI, -0.322 to 0.127; *Z*=-1.260, ns, respectively).

As shown in table 3 (Tab. 3), the results of the DID analysis indicated that “mutual support” and “communication” were associated with significantly higher scores in the online than in the face-to-face IPE program (0.194; 95% CI, 0.057 to 0.318; *Z*=3.035, *p*=.002, and 0.216; 95% CI, 0.062 to 0.337; *Z*=3.196, *p*=.001, respectively). Meanwhile, no significant differences were seen in the mean scores of the difference in “team structure” (-0.004; 95% CI, -0.118 to 0.128; *Z*=-0.426, ns), “leadership”, or “situation monitoring” (-0.057; 95% CI, -0.178 to 0.064; *Z*=-1.325, ns, and -.034; 95% CI, -0.162 to 0.088; *Z*=-0.905, ns, respectively) between the face-to-face and online IPE programs.

## 4. Discussion

The present results indicate that attitudinal effects in most categories, except for “mutual support” and “communication” in the T-TAQ instrument, were the same or more positive for the online IPE program. In particular, student attitudes toward “patient-centered care” in the modified ATHCTS and “team structure”, “leadership”, and “situation monitoring” in the T-TAQ changed significantly for the better to a similar extent. E-learning studies have been suggested to be effective in improving various professional competencies and attitudes [12], [13], and are known to play an important and complementary role in distance learning [14]. Some comparative studies have reported no differences in educational effects between academic outcomes in face-to-face and online learning [15], [16], [17], [18]. Therefore, online IPE programs may provide a similar effect for students learning collaborative practice for patient safety as a whole.

However, regarding attitudinal changes according to the modified ATHCTS, the mean score of the difference in “team efficiency” was significantly lower in the online than in the face-to-face IPE program according to the DID method. This corresponded well to the evidence that the mean of the regression factor scores for “team efficiency” in the online IPE program was significantly lower than that in the face-to-face program compared with the post-IPE stage. In addition, the mean score of the subscale itself did not change in the face-to-face IPE program, whereas it was significantly decreased in the online IPE program. These results indicate that the online IPE program reduced the perception of “team efficiency”, but the positive effect of the face-to-face IPE program remained. However, students learning via the online IPE program in the academic year 2020 experienced technological difficulties when accessing Moodle, Google, and Zoom. In online learning, technological difficulties are often a major disruptive factor, and can lead to a loss of the collegiality typically associated with face-to-face learning [39]. Some people may regard online learning as isolating compared with traditional learning methods because of the lack of the same opportunity for substantive social connection [40]. The shift from the traditional classroom-based approaches has caused some learners to feel isolated, while others have noted a lack of support from their online educators [41]. These findings suggest that online programs may significantly negatively impact learners’ attitudes with respect to “team efficiency” because of technological difficulties, which might be exacerbated by the lack of opportunity for social connection. These findings imply that the disadvantages associated with a student’s geographical characteristics can be mitigated by making the contents of e-learning tools easier for students to use.

Interestingly, regarding the attitudinal changes according to the T-TAQ, the mean score of the difference in “mutual support” was significantly higher in the online than in the face-to-face IPE program, as elucidated by the DID method. These findings corresponded well to the fact that the means of the regression factor scores for “mutual support” in the online program were significantly higher than those for the face-to-face program at the post-IPE stage. In addition, the mean score of the category did not change in response to the face-to-face IPE program, whereas the mean score in the online IPE program increased significantly. These results indicate that the online IPE program significantly improved student attitudes toward “mutual support”, but did not eliminate the negative effect of the face-to-face IPE program. Online IPE programs have been shown to lead to significant increases in student attitudes toward working in interprofessional teams before and after the COVID-19 pandemic [42], [43]. Studies on the effects of e-learning have found that this type of education can improve professional attitudes as one of the competencies [44]. The scenarios have been reported to be formative and summative, thereby “allowing participants to demonstrate team-based skills, including communication, mutual support, leadership, and situational monitoring” [45]. Early in student education, a common framework to describe the best practice model of interprofessional interactions must be developed. To identify the ideal timing of simulations in each program, curricular mapping has been performed to ensure sustainable curricular interaction and comparability in student clinical preparation for participation [46]. In addition, online IPE programs that utilize a case scenario may enhance the learning effects of mutual support, as well as clinical preparation for participation in learning the role of one’s own profession and those of others.

The mean score of the difference in “communication” was significantly higher in the online than in the face-to-face IPE program, as elucidated by the DID method. The change in the mean score itself was also similar to that of “mutual support”, indicating that the online IPE program significantly improved student attitudes toward “communication”, but did not eliminate the negative effects of the face-to-face IPE program. No significant improvement was identified in student attitudes regarding “communication” when implementing face-to-face IPE without patient safety components in 2018 [4]. The advantage of using e-learning methods is that they can foster a sense of collaborative community for participating learners [41]. The online environment has created vast opportunities for student–tutor interaction and has placed collaborative learning at the forefront of distance education [47]. Standardized patients can be trained as standardized family members to enhance student learning, particularly in communicating topics that are difficult to understand [48]. Therefore, online IPE programs may promote the communication attitude required for collaborative practice for patient safety to promote understanding the views of standardized patients and their families.

## 5. Conclusions

Online IPE programs appear to have a similar effect on students learning collaborative practice for patient safety as a whole. However, due to technological difficulties, online IPE programs may negatively impact the educational effects regarding attitudes toward “team efficiency”, and this may be exacerbated by a lack of opportunity for social connection. Meanwhile, online learning may distinctively improve attitudes toward “mutual support” by promoting understanding of the role of one’s own profession as well as those of others using a case scenario, in addition to the attitudes toward “communication” required for collaborative practice for patient safety. Overall, the present findings suggest that online IPE programs for patient safety may provide better education effects as a whole, although efforts to minimize technological difficulties will be necessary in the future.

## Notes

### Funding

This work was supported in part by a Grant-in-Aid for Scientific Research (to TM) from the Ministry of Education, Culture, Sports, Science and Technology of Japan (22K10626).

### Authors’ contributions

SN was responsible for conceptualization, investigation, methodology, analysis, writing the original draft and writing, reviewing, and editing subsequent drafts; TM was the Principal Investigator and responsible for conceptualization, investigation, methodology, analysis, writing the original draft and writing, reviewing, and editing subsequent drafts; BL, HM, and ES were responsible for conceptualization, investigation, data collection, reviewing, and editing; HS was involved in reviewing and editing; HW was responsible for conceptualization, investigation, methodology, writing the original draft, and writing, reviewing, and editing subsequent drafts, project administration, and supervision.

### Authors’ ORCIDs

- Takatoshi Makino: [0009-0003-9858-5827]
- Bumsuk Lee: [0000-0001-7508-6644]
- Hiroki Matsui: [0000-0003-3243-333X]
- Ena Sato: [0000-0002-7612-6115]
- Hiromitsu Shinozaki: [0000-0001-5525-3011]
- Hideomi Watanabe: [0000-0003-0571-3336]

## Acknowledgements

We wish to thank all the students who participated in the survey and the faculty of Gunma University for their cooperation with the data collection.

## Competing interests

The authors declare that they have no competing interests.

## Figures and Tables

**Table 1:**
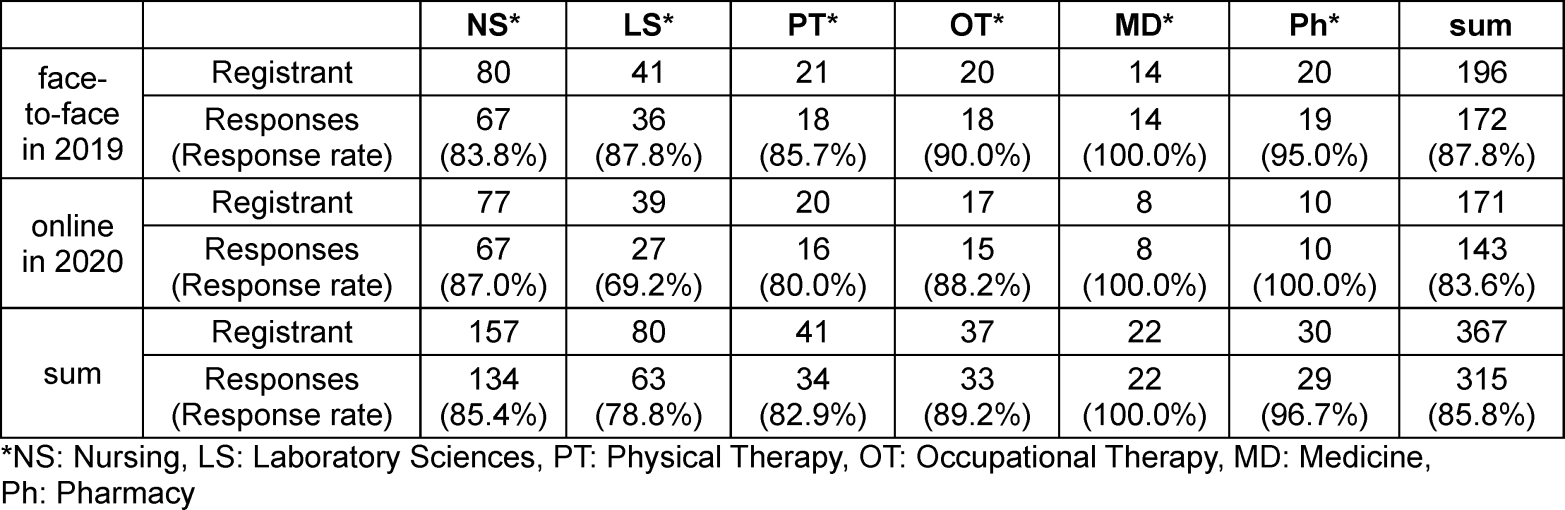
Responding sample demographics

**Table 2:**
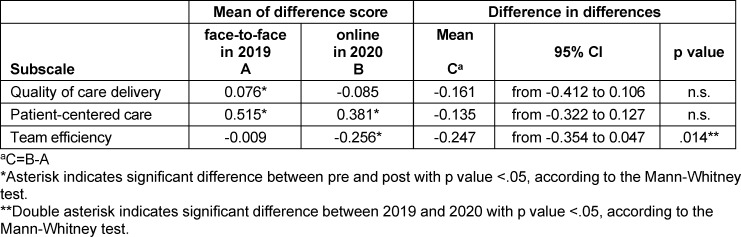
Difference in differences analysis of mean of difference score the modified ATHCTS between face-to-face in 2019 and online in 2020

**Table 3:**
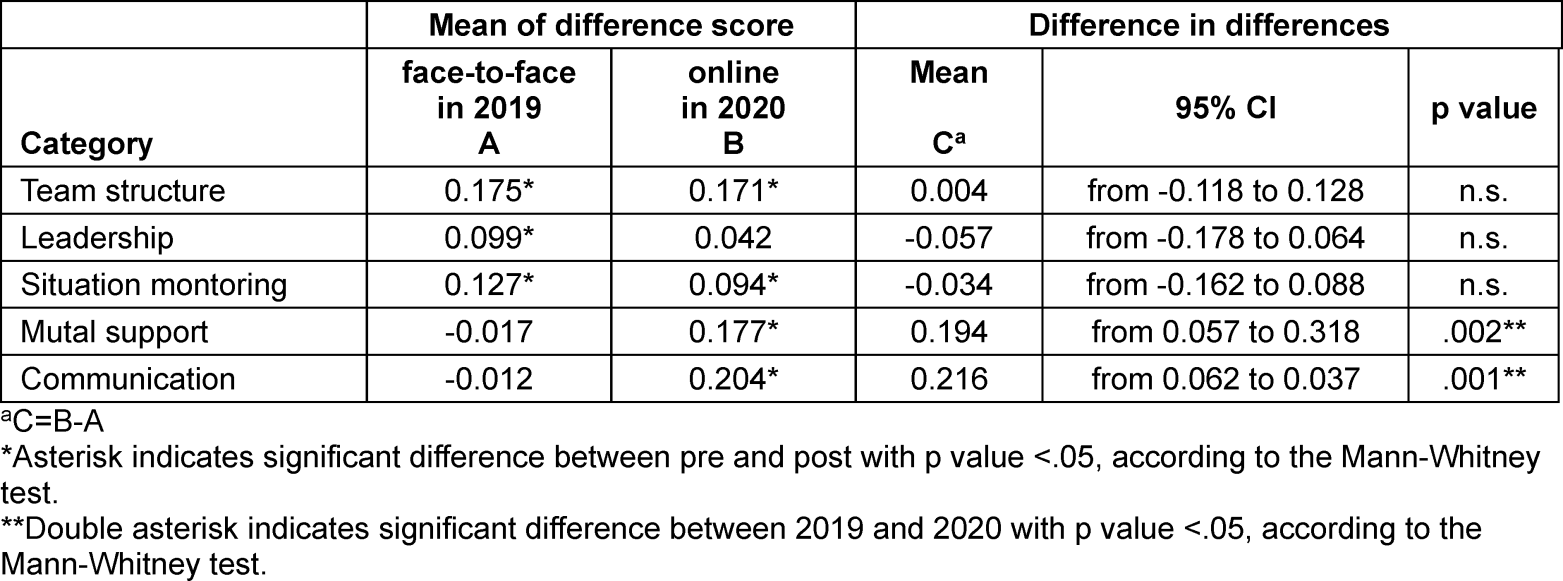
Difference in Differences analysis of mean of difference score of the T-TAQ between face-to-face in 2019 and online in 2020
